# Acute and Subacute Toxicity Studies of the Ethyl Acetate Soluble Proanthocyanidins of the Immature Inflorescence of *Cocos nucifera* L. in Female Wistar Rats

**DOI:** 10.1155/2019/8428304

**Published:** 2019-12-03

**Authors:** C. P. Ekanayake, M. G. Thammitiyagodage, S. Padumadasa, B. Seneviratne, C. Padumadasa, A. M. Abeysekera

**Affiliations:** ^1^Department of Chemistry, Faculty of Applied Sciences and Centre for Plant Materials and Herbal Products Research, University of Sri Jayewardenepura, Gangodawila, Nugegoda, Sri Lanka; ^2^Animal Centre, Medical Research Institute, Colombo 08, Sri Lanka; ^3^Department of Obstetrics and Gynaecology, Faculty of Medicine, University of Kelaniya, Sri Lanka; ^4^Department of Pathology, Faculty of Medical Sciences, University of Sri Jayewardenepura, Gangodawila, Nugegoda, Sri Lanka

## Abstract

Ayurvedic and traditional medical practitioners of Sri Lanka use the decoction of the immature inflorescence of *Cocos nucifera* L. (IC) variety aurantiaca for the treatment of menorrhagia. The progestogenic effect of the ethyl acetate soluble proanthocyanidins (EASPA) of the IC in female rats at a dose of 3.5 mg/kg body weight has been reported. Acute and subacute toxicity studies of EASPA of the IC carried out using female Wistar rats according to Organization for Economic Co-operation and Development (OECD) guidelines 423 and 407, respectively, are reported herein. In the acute toxicity study, a single dose of EASPA (2000 mg/kg body weight) was orally administered to rats, which were monitored for 14 days. In the subacute toxicity study, rats were orally administered with EASPA daily for 28 days at doses of 1.75, 3.5, 7, and 14 mg/kg body weight. No rat in either the acute or subacute toxicity study exhibited mortality or clinical signs of toxicity. Further, these rats did not show any significant change in their mean body weight, food, and water intake, haematological and biochemical parameters as well as in the results of their histopathological examinations compared to those of control group rats. According to results of the acute toxicity, the LD_50_ of EASPA is estimated to be greater than 2000 mg/kg body weight. Considering the results of the subacute toxicity study, the oral administration of EASPA daily for 28 days was well tolerated up to the dose, 14 mg/kg by rats. These results will be useful in the development of a novel therapeutic agent from EASPA of the IC for the treatment of menorrhagia, which incapacitates a considerable proportion of women worldwide.

## 1. Introduction

The coconut palm, *Cocos nucifera* L. is a member of the monocotyledonous family, Arecaceae and mainly grows in four major tropical coastal areas: Central and South America, Oceania, Southern Africa and Southern Asia including Sri Lanka [1, 2]. In Sri Lanka, it is classified into three varieties, Typica, Nana, and Aurantiaca. *Cocos nucifera* L. variety aurantiaca is commonly known as king coconut in English and “Thembili” in Sinhalese [3]. Ayurvedic and traditional medical practitioners of Sri Lanka use the decoction of the immature inflorescence of *Cocos nucifera* L. variety aurantiaca (IC) for the treatment of menorrhagia. Further, the use of the IC for the treatment of menorrhagia is mentioned in Ayurveda literature [4].

Preliminary phytochemical screening of the IC revealed that it contains a high amount of proanthocyanidins. The extraction, purification, and characterization of ethyl acetate soluble proanthocyanidins (EASPA) of the IC have previously been reported [5]. Furthermore, the effects of EASPA on oestrogen and progesterone levels in female rats have also been reported. The oral administration of EASPA to female rats for 28 days produced a significant increase in progesterone levels with no change in oestrogen levels in test group rats [6]. This finding is very significant as progestogens are widely used in the treatment of menorrhagia in Western medicine [7].

Medicinal plants are used for the prevention and treatment of various diseases worldwide. They are a rich source of diverse bioactive compounds that provide unlimited opportunities for the discovery of novel drugs and herbal remedies [8]. The selection of medicinal plant candidates for the discovery of new drugs and herbal remedies has mainly been facilitated by the wealth of knowledge stored in organized traditional medical systems around the world. Although a plethora of compounds from medicinal plants have been isolated and their pharmacological properties have been reported, only a small percentage of them have gone through drug development processes providing therapeutic drugs for clinical use. According to a study carried out in 2001 by Fabricant and Farnsworth, only a total of 122 distinct pure compounds from 94 plant species are reported to be in clinical use globally [9]. This scenario may be attributed to toxicity effects exhibited by such compounds and safety challenges encountered during the drug development process. Therefore, it is important to establish the safety of medicinal plants; their preparations and isolated compounds through well controlled and validated scientific protocols for the development of novel drugs and herbal remedies [10, 11]. Moreover, toxicity studies play a significant role in drug development procedure providing information on toxic doses and therapeutic indices of potential drugs. In this study, we report the acute and subacute toxicity studies of EASPA of the IC in female Wistar albino rats.

## 2. Materials and Methods

### 2.1. Chemicals and Reagent

All chemicals and solvents used for the study were of AR grade and purchased from Sigma-Aldrich, Germany. Commercially available test kits (ALP, ALT, glucose, cholesterol, and urea) purchased from Sigma-Aldrich, France were used to analyze biochemical parameters.

### 2.2. Instrumentation

Konelab 20XT auto analyzer analyzed biochemical parameters. Blood samples in the tubes were centrifuged by Harmonic series centrifuge. Light microscope (OLYMPUS CH-2) with ×4 and ×10 objective lens was used to examine the histopathological slides. Haematological parameters were analyzed using BCC 3000B, DIRUI three part Haematological analyzer. ^13^C NMR spectrum was obtained in deuterated methanol for purified EASPA with a Bruker Avance AV- 500 Nuclear Magnetic Resonance Spectrometer at 600 MHz.

### 2.3. Plant Materials

Fresh immature inflorescences of the healthy adult *Cocos nucifera *L. palms (variety aurantiaca) situated within the premises of University of Sri Jayewardenepura, Nugegoda, Sri Lanka were collected from January 2017 to April 2017. The immature inflorescence (the inflorescence situated just above the freshly opened inflorescence in the palm) was plucked and the spathe was removed before using them for the preparation of test material. They were identified and authenticated by Mr. I. U. Kariyawasam of Department of Botany and voucher specimen (Assess. No. A3 S13, 001) was deposited in the herbarium of the Department of Botany, Faculty of Applied Sciences, University of Sri Jayewardenepura, Sri Lanka.

### 2.4. Extraction, Purification, and Characterization of Ethyl Acetate Soluble Proanthocyanidins (EASPA) of the Immature Inflorescence of Cocos Nucifera L

Extraction and purification of EASPA of the IC was carried out according to previously published methods [5, 6, 12]. Evenly chopped fresh immature inflorescence (870.0 g) was extracted with 70% aqueous acetone containing 0.1% ascorbic acid under reflux for two hours. After cooling and filtration, the extract was saturated with sodium chloride. The acetone layer that salted out was removed and washed with the aqueous layer of sodium chloride-saturated 70% aqueous acetone containing 0.1% ascorbic acid. The resulting acetone layer was evaporated *in vacuo* at 45°C. The viscous residue obtained was then mixed with an equal volume of water and extracted with petroleum ether (40–60°C) followed by ethyl acetate. The combined ethyl acetate extracts were dried over anhydrous sodium sulphate and evaporated *in vacuo* at 45°C to produce the crude EASPA as a light brown powder (1.77 g).

Crude EASPA (1.00 g) was purified by column chromatography using Sephadex LH-20. The nonproanthocyanidin phenolics were eluted first with ethanol. Proanthocyanidin phenolics were then eluted with 70% aqueous acetone. Prussian blue and acid catalyzed cleavage tests were used to examine the collected fractions. Collected fractions, which gave positive results for both tests were combined. The acetone was removed *in vacuo* at 45°C and the resulting aqueous residue was freeze-dried to obtain purified EASPA (0.30 g) as an off white powder. Purified EASPA was characterized by ^13^C NMR spectroscopy.

### 2.5. Experimental Animals

Female Wistar albino rats (Species—*Rattus norvegicus*, Origin—CLEA Japan) approximately 12 weeks old, weighing 112 g–144 g were purchased from the Animal Center, Medical Research Institute (MRI), Sri Lanka for toxicity studies. Animals were housed in standard cages with sawdust as bedding and they were fed with a standard diet prepared according to a formula prescribed by Saboudry [13] and water *ad libitum*. Rats were exposed to a 12 hours light/dark cycle at room temperature. They were identified by color markings on their body. The rats were handled in accordance with the standard guidelines for the care and use of laboratory animals (CPCSEA guidelines). Rats were acclimatized to above-mentioned conditions for one week prior to the toxicity studies. Ethical approval was obtained from the Ethics Review Committee, Faculty of Medical Sciences, University of Sri Jayewardenepura, Nugegoda, Sri Lanka (Ref. No. 50/17) and necessary skills for handling rats were obtained from the Animal Center, Medical Research Institute (MRI), Sri Lanka under the supervision of the veterinarian before commencing the animal study.

### 2.6. Acute Oral Toxicity Study

Acute oral toxicity study of EASPA was performed using female Wistar rats according to the Organization for Economic Co-operation and Development (OECD) guideline 423 [14]. A total of 12 female rats were used for the study. Their weights were recorded and randomly divided into 2 groups (A and B), each containing 6 rats [15]. Tap water extract of EASPA/ tap water was orally administered to the rats under overnight fasting at a volume of 10 mL/kg body weight [14, 16]. Ethyl acetate soluble proanthocyanidins (EASPA) was dissolved in tap water and a single dose was administered at a dose of 2000 mg/kg body weight to group A rats. Group B served as the control group and the rats in this group were administered with tap water only. The rats of all groups were maintained under the same conditions with normal food and water. They were observed individually for the first critical 4 hours and thereafter twice daily (every day at 9.00 am and 3.00 pm) during the study period (14 days) for mortality, signs of toxicity (changes in the skin, fur, eyes, mucus membranes, respiratory depression) and behavioral changes (salivation, diarrhea, sleep, coma, lethargy). Further, body weight changes, food and water intake were also recorded during the study period.

The percentage of body weight change was calculated according to the following equation [10].

(1) $$\begin{array}{c} \text{Percentage body weight change}=\frac{\text{Body weight at the end of each week}-\text{Initial body weight}}{\text{Initial body weight}}\times 100. \end{array}$$

On the 14^th^ day, all the rats were kept fasting overnight. On the 15^th^ day, they were weighed and sacrificed by overdose inhalation of the anesthetic ether. Blood samples were collected by cardiac puncture for haematological and biochemical analyses. This was followed by histopathological studies.

#### 2.6.1. Biochemical Parameters and Haematology

Each blood sample (obtained under Section 2.6.) was divided into two separate tubes, with and without the anticoagulant, ethylenediamine–tetraacetate (EDTA). The blood samples in the tubes without the anticoagulant were kept for 2 hours until complete clotting was observed. Afterwards, tubes were centrifuged at 4000 rpm at 4°C for 10 minutes. Serum was then separated and subjected to analysis of biochemical parameters, alkaline phosphatase (ALP), alanine aminotransferase (ALT), urea, glucose, and cholesterol. Biochemical parameters were analyzed by standard methods using test kits with a Konelab auto analyzer. The blood samples in the tubes with the anticoagulant (EDTA) were immediately analyzed for haematological parameters (red blood count (RBC), haemoglobin (Hb), mean corpuscular volume (MCV), mean corpuscular haemoglobin (MCH), mean corpuscular haemoglobin concentration (MCHC), platelet count, white blood count (WBC), lymphocyte, monocyte, basophil, and neutrophil) using a haematological analyzer [17].

#### 2.6.2. Histopathological Studies

At the end of the acute toxicity study (on the 15^th^ day), all the rats in control and test groups were sacrificed by overdose inhalation of the anesthetic ether. Necropsy and gross examination on internal organs (liver, heart, kidney, uterus, and spleen) were carried out. The organs were observed macroscopically for any abnormalities and presence of lesions. Afterwards, the internal organs were dissected, cleaned of any fat and weighed to obtain the absolute weight of the organs. Relative organ weight (ROW) of different organs was calculated using the equation given below according to previously published methods [18, 19].

(2) $$\begin{array}{c} \text{ROW}=\frac{\text{Absoluteorganweight}\left(\text{g}\right)}{\text{Bodyweightofratonthedayofsacrifice}\;\left(\text{g}\right)}\times 100. \end{array}$$

Afterwards, organs were fixed in 10% buffered formalin solution and preserved for subsequent histopathological examinations. The preserved organs were taken out from the 10% buffered formalin solution and processed routinely and embedded in paraffin wax. Histology sections at a thickness of 4 *µ*m were obtained using a microtome. They were stained with hemotoxylin and eosin and observed under a light microscope (with × 40 objective lenses). A consultant histopathologist recorded pathological changes of the tissue sections in test groups in comparison to the control group and corresponding photomicrographs were taken [19].

### 2.7. Subacute Toxicity Study

Subacute toxicity study of EASPA was carried out using female Wistar rats according to the OECD guideline 407 [20]. A total of 30 female rats were used for the study. Their weights were recorded and randomly divided into 5 groups (P, Q, R, S, and T), each containing 6 rats. The rats were kept fasting overnight and tap water extract of EASPA/ tap water was orally administered to the rats at a volume of 10 mL/kg body weight, as similar to the acute toxicity study. EASPA was dissolved in tap water and rats in groups P, Q, R, and S were administered at dose levels of 1.75 mg/kg, 3.5 mg/kg (therapeutic dose), 7 mg/kg, and 14 mg/kg body weight, respectively, separately daily for 28 consecutive days. The therapeutic dose was calculated using the available knowledge on usage of IC in Ayurvedic medicine. The dose for rats was derived by extrapolating the human dose in milligrams according to the standard chart given in literature [21]. The resulting value was multiplied by the yield of purified EASPA to obtain the therapeutic dose. The tap water extract of EASPA was prepared on a daily basis. Rats in group T were kept as the control group and they were administered with tap water (vehicle) only. The rats of all groups were maintained under the same conditions with normal food and water. They were observed for mortality, signs of toxicity, and behavioral changes twice daily (every day at 9.00 am and 3.00 pm) for 28 days. Body weight changes, food, and water intake were also recorded from the onset of the study. On the 28^th^ day, all the rats were kept fasting overnight. On the 29^th^ day, they were weighed and sacrificed by an overdose inhalation of the anesthetic ether. Blood samples were collected by cardiac puncture for haematological and biochemical analyses. This was followed by histopathological studies.

#### 2.7.1. Biochemical Parameters and Haematology

At the end of the subacute toxicity study (on the 29^th^ day), rats in all groups who were kept fasting overnight, were weighed and sacrificed by an overdose inhalation of the anesthetic ether. Blood samples of rats in all groups were collected by cardiac puncture for haematological [red blood count (RBC), haemoglobin (Hb), mean corpuscular volume (MCV), mean corpuscular haemoglobin (MCH), mean corpuscular haemoglobin concentration (MCHC), platelet count, white blood count (WBC), lymphocyte, monocyte, basophil, and neutrophil) and biochemical (alkaline phosphatase (ALP), alanine aminotransferase (ALT), serum urea, serum glucose, and cholesterol] analyses, similar to the procedure described under Section 2.6.1.

#### 2.7.2. Histopathological Studies

On the 29^th^ day of the subacute toxicity study, all the rats in control and test groups were sacrificed using an overdose inhalation of the anesthetic ether and blood samples were collected by cardiac puncture method. Necropsy and gross examination of internal organs (liver, heart, kidney, uterus, and spleen) were carried out. Then, internal organs (liver, heart, kidney, uterus, and spleen) were dissected, cleaned of any fat, weighed, and observed macroscopically. Relative organ weight was also calculated. Finally, they were fixed in 10% buffered formalin solution for histopathological examination similar to the procedure described under Section 2.6.2.

### 2.8. Statistical Analysis

All the qualitative data were expressed as mean ± Standard Error of Mean (SEM). Every statistical analysis was performed with one-way analysis of variance (ANOVA) followed by the *t*-test using SPSS 16.0 software. Statistically significant differences were accepted at *p* ≤ 0.05.

## 3. Results

### 3.1. Extraction, Purification, and Characterization of EASPA

Extraction and purification of EASPA of the immature IC were carried out with minor modifications according to previously published methods. Crude proanthocyanidins extracted to aqueous acetone (70%) containing 0.1% ascorbic acid was partitioned into ethyl acetate to yield ethyl acetate soluble proanthocyanidins (EASPA) which after solvent removal yielded crude EASPA as a light brown powder in 0.20% by weight to the fresh inflorescence. In the purification of EASPA by Sephadex LH-20 column chromatography, nonproanthocyanidin phenolics were first eluted with ethanol and subsequently, EASPA that was adsorbed to Sephadex LH-20 was eluted with 70% aqueous acetone. All the fractions eluting from the column were analyzed for the presence of phenolics by the Prussian blue test and proanthocyanidins by the acid catalyzed cleavage test. Acetone fractions, which gave positive results for both the tests were combined and acetone was removed under reduced pressure. The resulting aqueous fraction upon freeze-drying yielded the purified EASPA as an off white powder in 0.063% by weight to the fresh inflorescence. The purified EASPA was characterized by ^13^C NMR spectroscopy. The ^13^C NMR spectrum was similar to what is reported previously and showed signals characteristic for epicatechin units indicating that EASPA is composed mainly of epicatechin units [5] and was used for toxicity studies.

### 3.2. Acute Toxicity Study

**Figure 1:**
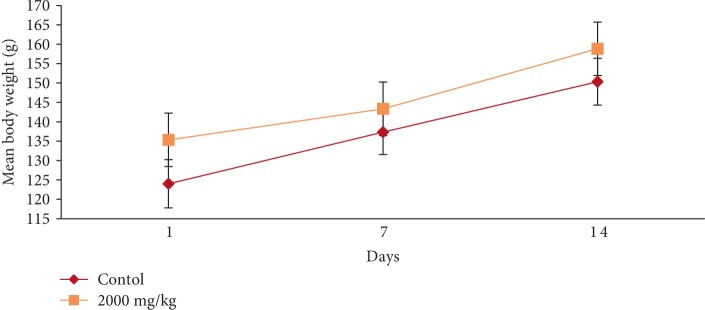
Effect of EASPA on mean body weight in acute toxicity study. Values are expressed as a mean. The error bars represent the standard error of the mean (*n* = 6 and *p* < 0.05).

**Figure 2:**
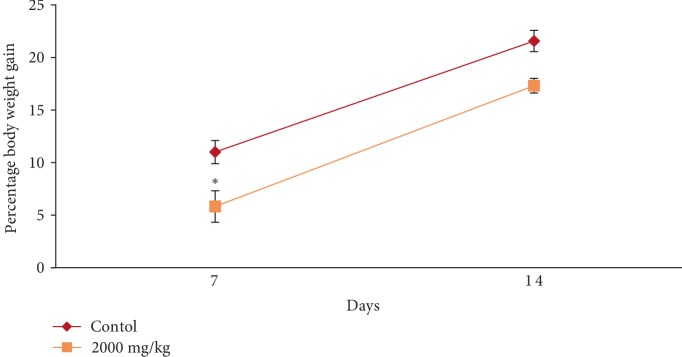
Effect of EASPA on percentage body weight gain in acute toxicity study. Values are expressed as a mean. The error bars represent the standard error of the mean (*n* = 6,*p* < 0.05 and ^∗^ = significant difference from the control group).

In the acute toxicity study, single oral administration of EASPA at the dose level of 2000 mg/kg body weight to rats did not result in any mortality. Any of the treated rats did not exhibit any visible signs of toxicity or behavioral changes and were found to be normal throughout the 14-day study period similar to rats of the control group. Mean body weight of test and control group rats are given in Figure 1. There was a gradual increase in the mean body weight of treated and control group rats during the study period. There was no statistical difference in mean body weight of rats between treated and control groups from the onset up to the end of the study period (*p* > 0.05). The percentage body weight gain of treated rats at the dose level of 2000 mg/kg body weight was similar (*p* > 0.05) to that of the control group rats at the end of the study period. However, the percentage body weight gain of treated rats was significantly lower (*p* < 0.05) than that of the control group rats after the first week (Figure 2). Although the food consumption of treated rats was similar (*p* > 0.05) to that of the control group rats at the end of the study period the consumption of food by rats treated with EASPA at the dose of 2000 mg/kg body weight was significantly lower (*p* < 0.05) than that of the control group rats at the end of the first week. The water intake by treated rats was similar (*p* > 0.05) to that of the control group rats during the study period. Haematological and biochemical parameters determined at the end of the 14-day study period of rats treated with EASPA at a single dose of 2000 mg/kg body weight compared to those of the control group rats are presented in Tables 1 and 2, respectively. According to the results, haematological parameters such as total red blood cell count (RBC), total white blood cell count (WBC), platelet count, haemoglobin, mean corpuscular haemoglobin (MCH), and mean corpuscular haemoglobin concentration (MCHC) and biochemical parameters such as serum urea, alkaline phosphatase (ALP), alanine aminotransferase (ALT), glucose and cholesterol levels of treated rats were similar to those of the control group rats (*p* > 0.05). Gross examination of vital internal organs during necropsy at the end of the study period of treated rats revealed no abnormalities in the color or texture in comparison to that of control group rats. Relative organ weights (ROW) of test group rats compared to that of control group rats are shown in Table 3. There was no significant difference in ROW between test group and control group rats. Light microscopic examination of vital organs including liver, kidney, heart, spleen, and uterus of treated rats did not show any gross pathological lesions and was similar to that of control group rats.

### 3.3. Subacute Toxicity Study

#### 3.3.1. Mortality, General Signs of Toxicity, and Food and Water Consumption of Rats

**Figure 3:**
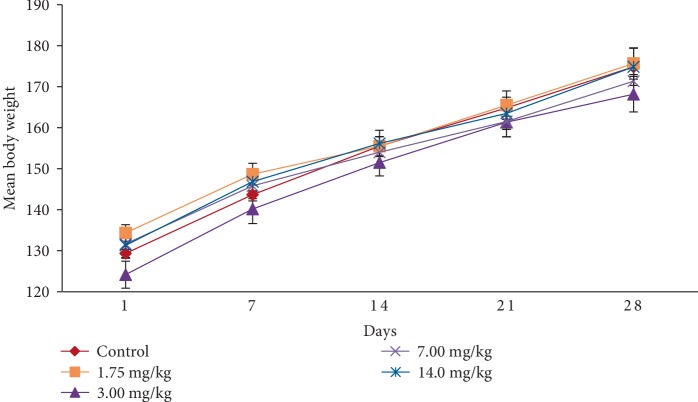
Effect of EASPA on mean body weight in the subacute toxicity study. Values are expressed as a mean. The error bars represent the standard error of the mean (*n* = 6 and *p* < 0.05).

**Figure 4:**
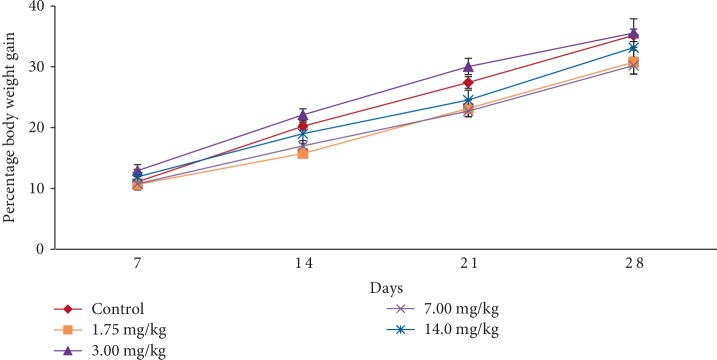
Effect of EASPA on percentage body weight gain in the subacute toxicity study. Values are expressed as a mean. The error bars represent the standard error of the mean (*n* = 6 and *p* < 0.05).

**Figure 5:**
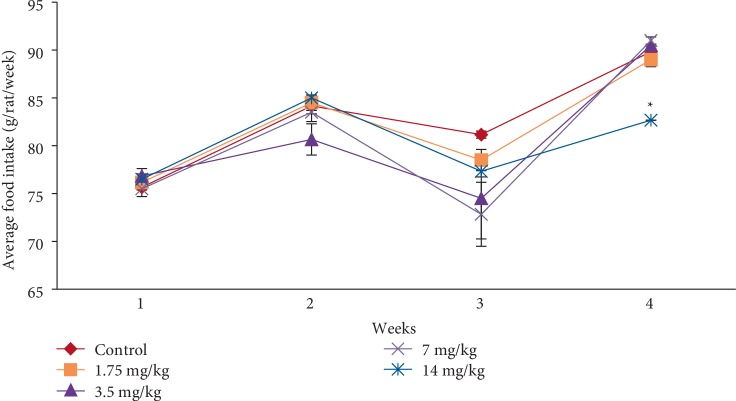
Effect of EASPA on average food intake in the subacute toxicity study. Values are expressed as a mean. The error bars represent the standard error of the mean (*n* = 6 and *p* < 0.05 and ^∗^ = significant differences from the control group).

**Figure 6:**
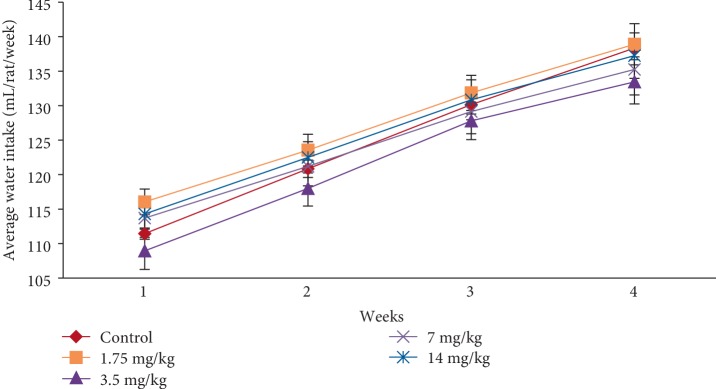
Effect of EASPA on average daily water intake (mL/rat/week) in the subacute toxicity study. Values are expressed as a mean. The error bars represent the standard error of the mean (*n* = 6 and *p* < 0.05).

In the subacute toxicity study, oral administration of EASPA daily for 28 days at doses of 1.75 mg/kg, 3.5 mg/kg, 7 mg/kg, and 14 mg/kg body weight to rats did not result in any mortality. Rats of any of the test groups did not show any visible signs of toxicity such as changes in skin, eyes, fur, and mucous membranes. Further, these rats did not show any behavioral changes including salivation, sleep, coma, lethargy, and diarrhea and were found to be normal throughout the study period compared to the control group. The effect of EASPA on the mean body weight of rats in all test groups is given in Figure 3. According to the results, a gradual increase in the mean body weight was observed in rats in treated and control groups during the 28-day study period. There was no statistical difference in the mean body weight of rats between treated and control groups from the onset up to the end of the study period (*p* > 0.05). Similarly, there was no significant difference in the percentage body weight gain between treated and control group rats at the end of 7, 14, 21, and 28 days (*p* > 0.05) (Figure 4). Generally, weekly food consumption of treated rats was similar to that of control group rats during the study period (*p* > 0.05) (Figure 5). However a significant decrease in food consumption was observed during the 4^th^ week in rats of the group treated with EASPA at the dose of 14 mg/kg body weight (*p* < 0.05) compared to the control group. The water consumption, which was also recorded throughout the study period, was found to be similar for rats in all treated and control groups (*p* > 0.05) (Figure 6).

#### 3.3.2. Biochemical Parameters and Haematology

The effect of oral administration of EASPA daily for 28 days on haematological and biochemical parameters which were determined at the end of the study period in treated and control group rats are summarized in Tables 4 and 5, respectively. The haematological parameters of rats of all treated groups were similar to those of control group rats, with the exception of mean corpuscular haemoglobin (MCH) and mean corpuscular haemoglobin concentration (MCHC) of test group rats treated with EASPA at the dose level of 14 mg/kg body weight) (*p* > 0.05). There was a significant increase, although slight in mean corpuscular haemoglobin (MCH) and mean corpuscular haemoglobin concentration (MCHC) of rats of the group treated with EASPA at dose of 14 mg/kg body weight (*p* < 0.05) compared to the control group rats. When considering biochemical parameters (serum urea, alkaline phosphatase (ALP), alanine aminotransferase (ALT), glucose, and cholesterol), there was no significant difference between treated and control group rats (*p* > 0.05).

#### 3.3.3. Macropathology and Relative Organ Weight (ROW) of Rats

Vital internal organs such as liver, kidney, spleen, heart, and uterus in rats of all test groups did not show any abnormality in color and texture compared to those of control group rats in gross examinations during necropsy at the end of the study period. The ROW of dissected organs (liver, kidney, spleen, heart, and uterus) in treated and control group rats which was recorded during necropsy is shown in Table 6. According to results, the ROW of treated rats in all test groups was similar to that of the control group rats (*p* > 0.05).

### 3.3.4. Histopathological Studies

**Figure 7:**
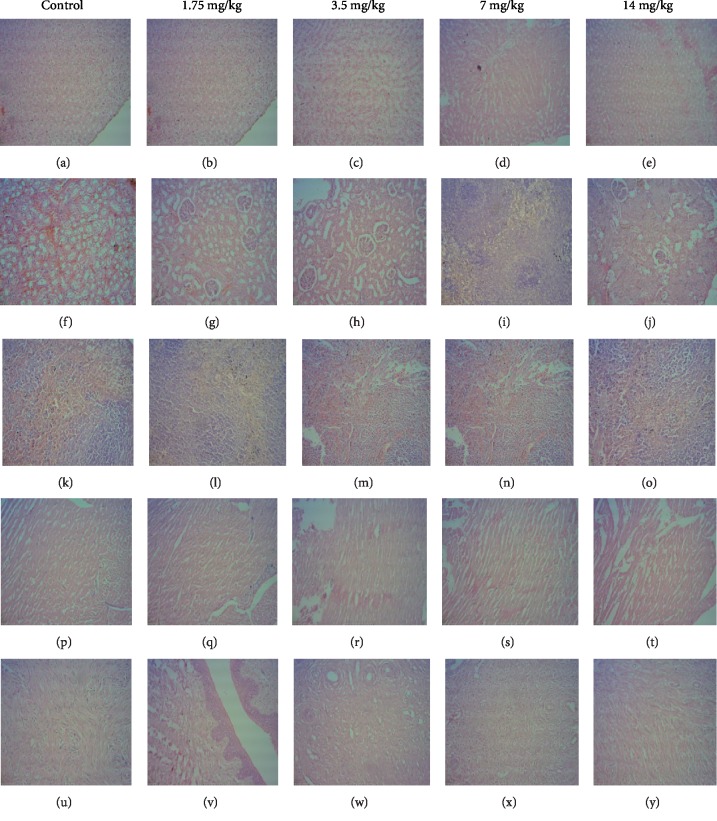
Effect of EASPA on histomorphologies of vital organs in test group rats in subacute toxicity study (H and E Stain, 100x). [(a), (b), (c), (d), (e): liver; (f), (g), (h), (i), (j): kidney; (k), (l), (m), (n), (o): spleen; (p), (q), (r), (s), (t): heart; (u), (v), (w), (x), (y): uterus].

Light microscopic examinations of histopathological sections of vital organs (liver, kidney, heart, spleen, and uterus) in test and control group rats are shown in Figure 7. Histopathological evaluation of tissue sections of vital organs in rats of all test groups showed a normal morphological architecture without any treatment related pathological changes and was similar to that of control group rats.

## 4. Discussion

Practitioners in Ayurvedic and traditional medicine use the IC for the treatment of menorrhagia in Sri Lanka. According to preliminary phytochemical investigation, the IC contains a high amount of proanthocyanidins. The extraction, purification, and characterization of EASPA of the IC have previously been reported [5]. The ^I3^C NMR spectrum of EASPA in this case was similar to that of previously reported and indicated its purity to be used for toxicity studies. Furthermore, effects of EASPA on oestrogen and progesterone levels in female rats have also been reported. The oral administration of EASPA at the dose of 3.5 mg/kg body weight in female rats for 28 days produced a significant increase in progesterone levels with no change in oestrogen levels in test group rats [6]. The dose of 3.5 mg/kg body weight, which is the therapeutic dose was calculated using the available knowledge on usage of IC in Ayurvedic medicine. The dose for rats was derived by extrapolating the human dose in milligrams according to the standard chart given in literature [21]. This biological activity of EASPA is significant as progestogens are being used in Western medicine for the treatment of menorrhagia. Toxicity studies are important in drug development providing information on toxic doses and therapeutic indices of potential drugs. Therefore, the present study was undertaken to evaluate acute and subacute toxicities of the EASPA using a rat model.

Generally, a higher dose of the test material is selected for the acute toxicity study. In this study, limit dose, 2000 mg/kg body weight was selected as described in the OECD guideline 423 [14]. A subacute toxicity study was performed according to the OECD guideline 407. In this study, the highest dose, 14 mg/kg body weight which is 4x therapeutic dose, was selected with the aim of inducing toxic effects, but not death or severe suffering. Thereafter, two-fold intervals of the highest dose were selected for setting off other descending dose levels [20].

In acute toxicity study, rats treated at the dose level of 2000 mg/kg body weight did not reveal any mortality, visible signs of toxicity or behavioral changes similar to that of control group rats during the 14-day study period. Similar observations were made for rats of all four treated groups in the subacute toxicity study during the 28-day study period. Mortality and clinical signs of toxicity are not only important observations in toxicity studies but also indicators of toxicity effects induced by the test material [22].

Body weight change is a sensitive indication of the general health status of animals [23]. Body weight loss of animals more than 20% is considered as critical and according to CACC and OECD guidelines, this incident is defined as one of the human end points [24, 25]. In the acute toxicity study, there was no significant difference in mean body weight of rats treated at the dose level 2000 mg/kg body weight compared to the control group rats during the study period. The percentage body weight gain and the food intake of treated rats at the dose level of 2000 mg/kg body weight were similar to those of the control group rats at the end of the study period. Although, the treated rats did not show any body weight loss, the percentage body weight gain and the food intake of these treated rats were significantly lower than those of the control group rats after the first week. However, these rats did not show any body weight loss after the first week. In animal nutrition, proanthocyanidins have been considered as antinutrients due to their astringency and ability to bind with several micronutrients, thereby reducing digestion and absorption of food [26, 27]. Low food intake of rats treated at the level of 2000 mg/kg body weight may be attributed to the astringent property of EASPA [28]. Therefore, it could be argued that low food intake of these rats may have contributed to their low percentage body weight gain during the first week, compared to the control group rats. In the subacute toxicity study, the mean body weight and the percentage body weight gain of rats in all test groups were similar to those of the control group rats at the end of 7, 14, 21, and 28 days. Therefore, it can be suggested that the oral administration of EASPA to rats daily for 28 days at the above-mentioned dose levels did not produce any treatment related body weight change during the study period. Generally, there was no significant difference in weekly food intake of these rats during the study period. However, a significant decrease in food consumption was observed during the fourth week in rats treated with EASPA at the dose level of 14 mg/kg body weight. This observation may not be toxicologically significant, as the reduced food intake of these rats had no effect on their body weight. According to observed results, it reveals that the basic metabolic processes of rats of all test groups in the subacute toxicity study were not adversely affected at tested doses.

In toxicity studies, serum biochemistry analyses play a major role in evaluating the possible toxic effects induced by the oral treatment of the test material [29, 30]. There was no significant difference in biochemical parameters in rats of all test groups compared to the control group in both acute and subacute toxicity studies. Serum biochemical parameters are important in analysis of liver and kidney functions in toxic evaluation of test materials as these organs are necessary for the survival of an organism and they metabolize and detoxify the organism from xenobiotic and endogenous compounds [31]. The liver is the major site of drug metabolism. It is considered as the site of cholesterol synthesis, degradation, and disposal. The liver plays a key role in synthesis of glucose and it generates free glucose from hepatic glycogen stores [32]. According to results observed it is possible to suggest that EASPA has no effect on lipid and carbohydrate metabolism since no significant changes were observed in glucose and cholesterol levels in rats of all treated groups in both toxicity studies. Liver function tests such as serum alanine aminotransferase (ALT) and serum alkaline phosphatase (ALP) can be used to predict liver malfunction in toxicity studies. Elevated levels of ALP and ALT are indicative of liver disease or hepatotoxicity [33]. The levels of ALP and ALT in rats of all test groups in both acute and subacute toxicity studies were similar to those of the control group rats. From these results it is possible to suggest that EASPA does not cause any acute toxicity effects in rats at the dose level of 2000 mg/kg body weight and subacute toxicity effects in rats at dose levels of 1.75, 3.5, 7, and 14 mg/kg body weight on the function of hepatocytes. Serum urea level of rats can be used to assess renal dysfunction in toxicity studies. Nonsignificant changes observed in serum urea level in rats of all test groups in both toxicity studies may suggest that EASPA does not alter the normal kidney function of these rats.

In toxicity studies, haematology analyses also play a major role in evaluating the possible toxic effects induced by the oral treatment of the test material [29, 30]. Bone marrow is considered as one of the most sensitive targets of toxic compounds. The status of bone marrow activity and intravascular system of treated rats can be monitored by their haematological parameters [34]. Further, changes in the haematological system of treated rats have a higher predictive value for toxicity in humans compared to animals when data is extrapolated from animal studies [35]. In the acute toxicity study, all haematological parameters in rats treated with EASPA at the dose level of 2000 mg/kg body weight were similar to those of the control group rats. In the subacute toxicity study, all haematological parameters in rats of all treated groups excluding mean corpuscular haemoglobin (MCH) and mean corpuscular haemoglobin concentration (MCHC) of the test group rats treated with EASPA at the dose level of 14 mg/kg body weight, were similar to those of the control group rats. Studies by Abrar et al. have reported an improvement in red blood cell count (RBC) and erythrocyte indices (MCH, MCHC) following oral treatment of grape seed proanthocyanidins in rabbits [36]. However, in our study, a significant increase of two erythrocyte indices in rats treated at the dose level of 14 mg/kg body weight is not accompanied with an increase of RBC. Therefore, this observation may not be treatment related, but rather incidental [37]. Considering nonsignificant changes in all haematological parameters of treated rats compared to the control group rats in both toxicity studies, it is possible to suggest that the oral treatment of EASPA is nontoxic to haematological parameters.

The liver, kidney, spleen, heart, and uterus are primary organs, which are affected by metabolic reactions caused by toxic compounds [38]. The color, texture and hypertrophy of these internal organs are some of the initial indications of organ toxicity, induced by toxic compounds. Macroscopic examination of internal organs in rats of all test groups in both acute and sub acute toxicity studies did not show any changes in color and texture compared to the control group rats during necropsy. Further, no hypertrophy was observed in internal organs of these rats. Organ weight is also a significant marker, which can be used to determine physiological and pathological status of animals. The ROW of treated rats is one of the fundamental markers used to confirm any treatment related injury in their internal organs [39]. Since there was no significant change in ROW of internal organs such as liver, kidney, spleen, heart, and uterus in rats of all test groups compared to that of control group rats in both toxicity studies, it is possible to suggest that the oral treatment of EASPA to rats do not produce toxic effects in them. Histopathological examinations of internal organs such as liver, kidney, spleen, heart, and uterus of rats in all test groups in both toxicity studies showed a normal cellular architecture and were similar to those of the control group rats. Sections of the liver of these rats showed no evidence of cellular injury, chlolestasis or cell necrosis and the arrangement of hepatocytes and lobular architecture was normal. Histopathological sections of renal tissues of these rats showed a normal architecture with no evidence of glomerulosclerosis, interstitial inflammation or parenchymal scarring. However, few histological slides of renal tissue in rats of the test group treated with EASPA at dose level of 14 mg/kg body weight in the subacute toxicity study showed mild swelling of tubular epithelial cells, indicative of early, reversible tubular injury. Histopathology sections of heart muscle in all treated rats in both acute and subacute toxicity studies did not show any features of myocardial necrosis. Histopathological sections of the spleen of these rats showed no abnormality other than mild congestion. Endometrial and myometrial sections of uteri of all treated rats were within normal histological limits. These observations were supported and confirmed by nonsignificant changes in biochemical parameters of all treated rats in both toxicity studies indicating that the oral treatment of EASPA to these rats did not induce significant detrimental changes and morphological alterations in their internal organs. However, in the subacute toxicity study, oral treatment of EASPA at the dose level of 14 mg/kg body weight to rats of the test group seems to have induced a mild toxicity in kidneys.

Considering the results of the acute toxicity study, it is possible to suggest that a single oral administration of EASPA to rats was well tolerated up to the dose level of 2000 mg/kg body weight. Therefore, it is possible to suggest that the LD_50_ of EASPA is above 2000 mg/kg body weight via oral route. According to the Globally Harmonized System of Classification and Labeling of Chemicals under OECD guideline, 423, EASPA can be classified into the category 5 (LD_50_ > 2000 mg/kg), which was the lowest toxicity class in the classification. According to results of the subacute toxicity study, the oral administration of EASPA to rats daily for 28 days at 1.75, 3.5, 7, and 14 mg/kg body weight dose levels is safe.

## 5. Conclusion

This study provides valuable data on acute and subacute toxicity studies of EASPA of the immature inflorescence of *Cocos nucifara* L. variety aurantiaca. Since there were no deaths or signs of toxicity in treated rats during the acute toxicity study, it is possible to suggest that the LD_50_ of EASPA is greater than 2000 mg/kg body weight via oral route. Observations made during the subacute toxicity study suggest that the long term intake (28-days) of EASPA at tested dose levels including the therapeutic dose do not induce any toxic effects in treated rats in comparison to control group rats. Thus, oral treatment of EASPA to rats has a wide margin of safety and potential for development of a novel therapeutic agent for the treatment of menorrhagia, which incapacitates a considerable proportion of women worldwide.

## Figures and Tables

**Table 1:** Effect of EASPA on serum haematological parameters in acute toxicity study.

Parameters	Unit	Control	2000 mg/kg
WBC	10^9^/L	6.10 ± 1.06	6.15 ± 0.53
RBC	10^12^/L	7.48 ± 0.42	7.08 ± 0.08
Haemoglobin	g/dL	15.35 ± 0.83	14.45 ± 0.07
Haematocrit	%	43.2 ± 2.78	41.7 ± 0.42
MCV	fL	117.55 ± 0.72	117.9 ± 0.69
MCH	pg	20.0 ± 0.07	20.45 ± 0.32
MCHC	g/dL	34.1 ± 0.48	34.62 ± 0.62
PLT	10^9^/L	912.25 ± 67.43	908.5 ± 48.73
Lymphocytes	10^9^/L	5.65 ± 0.98	5.65 ± 0.50
Monocytes	10^9^/L	0.3 ± 0.06	0.3 ± 0.03
Granules	10^9^/L	0.15 ± 0.03	0.2 ± 0.00
RDW-CV	%	12.27 ± 0.23	12.4 ± 0.06
MPV	fL	12.0 ± 0.28	11.8 ± 0.33
PCT	%	0.57 ± 0.04	0.54 ±0.04

Values are expressed as a mean ± SEM (*n* = 6 and *p* < 0.05).

WBC: Total white blood cell count; RBC: total red blood cell count; PLT: platelet count; MCV: mean corpuscular volume; MCH: mean corpuscular haemoglobin; MCHC: mean corpuscular haemoglobin concentration; RDW-CV: red blood cell distribution width; MPV: mean platelet volume; PCT: procalcitonin.

**Table 2:** Effect of EASPA on serum biochemical parameters in acute toxicity study.

Group	Unit	Control	2000 mg/kg
ALP	U/L	229.25 ± 19.78	245.67 ± 25.94
ALT	U/L	45.125 ± 0.63	93.93 ± 34.57
Glucose	mg/dL	91.46 ± 12.11	56.42 ± 7.26
Cholesterol	mg/dL	50.82 ± 1.27	48.96 ± 1.34
Urea	mmol/L	3.55 ± 0.16	3.23 ± 0.20

Values are expressed as a mean ± SEM (*n* = 6 and *p* < 0.05).

**Table 3:** Effect of EASPA on relative organ weight in acute toxicity study.

Organ	Control	2000 mg/kg
Liver	4.44 ± 0.03	4.39 ± 0.03
Kidneys	0.40 ± 0.02	0.42 ± 0.00
Heart	0.34 ± 0.01	0.35 ± 00
Spleen	0.29 ± 0.00	0.30 ± 0.00
Uterus	0.65 ± 0.02	0.71 ± 0.04

Values are expressed as a mean ± SEM (*n* = 6 and *p* < 0.05).

**Table 4:** Effect of EASPA on serum haematological parameters in the subacute toxicity study.

Parameters	Unit	Control	1.75 mg/kg	3.5 mg/kg	7 mg/kg	14 mg/kg
WBC	10^9^/L	6.20 ± 1.61	5.60 ± 0.56	6.40 ± 0.53	7.20 ± 0.35	5.50 ± 0.04
RBC	10^12^/L	6.12 ± 0.32	6.89 ± 0.08	6.24 ± 0.21	6.66 ± 0.10	6.20 ± 0.13
Haemoglobin	g/dL	11.70 ± 0.37	12.80 ± 0.68	12.90 ± 0.49	13.40 ± 0.17	13.00 ± 0.08
Haematocrit	%	33.10 ± 1.46	37.00 ± 0.47	34.90 ± 1.47	35.90 ± 0.40	34.60 ± 0.71
MCV	fL	109.43 ± 0.78	107.45 ± 0.49	111.70 ± 0.93	107.80 ± 0.44	111.60 ± 0.17
MCH	pg	19.55 ± 0.86	20.10 ± 0.14	20.30 ± 0.28	20.20 ± 0.08	21.00 ± 0.62^∗^
MCHC	g/dL	35.8 ± 1.25	37.42 ± 0.53	36.95 ± 0.31	37.3 ± 0.17	37.65 ± 1.02^∗^
PLT	10^9^/L	558.07 ± 109.03	495.33 ±74.24	522.00 ± 10.7	431.00 ± 69.3	535.00 ± 17.4
Lymphocytes	10^9^/L	5.5 ± 0.20	5.87 ± 0.21	5.90 ± 0.49	5.70 ± 0.45	5.1.00 ± 0.04
Monocytes	10^9^/L	0.37 ± 0.06	0.40 ± 0.05	0.30 ± 0.04	0.30 ± 0.04	0.20 ± 0.00
Granules	10^9^/L	0.20 ± 0.00	0.26 ± 0.02	0.20 ± 0.00	0.20 ± 0.00	0.20 ± 0.00
RDW-CV	%	23.90 ± 0.54	23.75 ± 0.19	23.40 ± 0.26	23.70 ± 0.31	23.30 ± 0.13
MPV	fL	10.60 ± 0.12	10.40 ± 0.20	10.00 ± 0.26	10.00 ± 0.08	10.10 ± 0.13
PCT	%	0.30 ± 0.05	0.25 ± 0.03	0.26 ± 0.01	0.21 ± 0.03	0.26 ± 0.00

Values are expressed as a mean ± SEM (*n* = 6, *p* < 0.05, and ^∗^ = significant difference from the control group).

RBC: Total red blood cell count; WBC: total white blood cell count; PLT: platelet count; MCV: mean corpuscular volume; MCH: mean corpuscular haemoglobin; MCHC: mean corpuscular haemoglobin concentration; RDW-CV: red blood cell distribution width; MPV: mean platelet volume; PCT: procalcitonin.

**Table 5:** Effect of EASPA on serum biochemical parameters in the subacute toxicity study.

Biochemical parameters	Unit	Control	1.75 mg/kg	3.5 mg/kg	7 mg/kg	14 mg/kg
ALP	U/L	226.83 ±15.14	246.50 ± 9.75	245.17 ± 3.00	244.00 ± 3.96	260.83 ± 5.59
ALT	U/L	72.31 ± 3.26	68.35 ± 4.05	70.08 ± 10.00	74.90 ± 12.23	64.55 ± 8.07
Glucose	mg/dL	75.24 ± 10.03	80.9 ± 10.28	83.12 ± 22.28	74.38 ± 7.48	82.06 ± 5.05
Cholesterol	mg/dL	62.95 ± 3.56	63.51 ± 1.78	64.16 ± 2.13	55.03 ± 0.99	60.08 ± 0.77
Urea	mmol/L	7.03 ± 0.48	5.91 ± 0.16	6.03 ± 0.14	6.32 ± 0.23	5.95 ± 0.23

Values are expressed as a mean ± SEM (*n* = 6 and *p* < 0.05). (ALP: Serum alkaline phosphatase; ALT: serum alanine aminotransferase.)

**Table 6:** Effect of EASPA on relative organ weights in the subacute toxicity study.

Group	Liver	Kidneys	Heart	Spleen	Uterus
Control	4.63 ± 0.13	0.79 ± 0.00	0.37 ± 0.01	0.25 ± 0.00	0.91 ± 0.03
1.75 mg/kg	4.49 ± 0.15	0.79 ± 0.02	0.34 ± 0.01	0.24 ± 0.00	0.83 ± 0.05
3.5 mg/kg	4.53 ± 0.15	0.8 ± 0.00	0.35 ± 0.01	0.23 ± 0.00	0.92 ± 0.00
7 mg/kg	4.40 ± 0.28	0.82 ± 0.02	0.34 ± 0.01	0.25 ± 0.01	0.98 ± 0.00
14 mg/kg	3.42 ± 0.29	0.73 ± 0.01	0.35 ± 0.00	0.23 ± 0.00	0.82 ± 0.02

Values are expressed as a mean ± SEM (*n* = 6 and *p* < 0.05).

## Data Availability

The data used to support the findings of this study are included within the article. The ^13^C NMR spectrum of EASPA is available on request.
